# Efficacy of anterior serratus plane block and intercostal nerve block in cardiothoracic surgery: a meta-analysis

**DOI:** 10.3389/fsurg.2026.1749519

**Published:** 2026-03-11

**Authors:** Tao Yuan, Meiyuan Pan, Yihan Luo, Dengke Duan, Shangdao Lai

**Affiliations:** Meizhou School of Clinical Medicine, Guangdong Medical University (Meizhou People’s Hospital), Meizhou, China

**Keywords:** cardiothoracic surgery, intercostal nerve block, meta-analysis, serratus anterior plane block, systematic review

## Abstract

**Background:**

This study aims to evaluate the differences in analgesic efficacy between the SAPB and INB in cardiothoracic surgery through a meta-analysis.

**Methods:**

PubMed, Cochrane Library, Embase, and Web of Science were searched from the establishment of the databases until July 10, 2025. All randomized controlled trials (RCTs) comparing the efficacy of SAPB and INB in cardiothoracic surgery were included. Quality assessment was performed using risk of bias. All data were analyzed using Stata 15 software.

**Results:**

A total of 9 randomized controlled trials involving 606 patients were included, meta-analysis results indicated that no differences in 6-h pain scores [SMD = 0.28, 95% CI (−0.50, 1.06)], 12-h pain scores [SMD = −0.59, 95% CI (−1.71, 0.53)], 24-h pain scores [SMD = −0.07, 95% CI (−0.67, 0.52)], incidence of nausea and vomiting[RR = 0.84, 95% CI (0.27, 2.57)] and length of hospital stay [SMD = 0.01, 95% CI (−0.30, 0.32)]between the SAPB group and the INB group, However, compared with INB, SAPB may reduce total opioid consumption[SMD = −1.99, 95% CI (−3.21, −0.77)].

**Conclusions:**

Overall, current evidence suggests that SAPB provides analgesic efficacy comparable to INB in cardiothoracic surgery. Subgroup analyses indicated that SAPB may be associated with lower pain scores in thoracotomy procedures and reduced opioid consumption in certain clinical settings; however, these findings should be interpreted cautiously due to heterogeneity across studies. Further high-quality randomized controlled trials are warranted to confirm these results.

**Systematic Review Registration:**

https://www.crd.york.ac.uk/PROSPERO/view/CRD420251080642, PROSPERO CRD420251080642.

## Background

In cardiothoracic surgery, postoperative pain management remains a challenging clinical issue. Traditional pain relief methods include pharmacological treatment and nerve block techniques (1, 2). With advancements in surgical techniques, local anesthesia and nerve blocks have become important tools for managing postoperative pain (3). However, traditional intercostal nerve block (INB) has certain technical limitations and variability in analgesic duration (4). Therefore, optimizing postoperative analgesic strategies remains an important clinical objective (5).

Serratus anterior plane block (SAPB) is a relatively new nerve block technique that has been widely applied in cardiothoracic surgery in recent years (6). This method involves injecting anesthetic drugs into the space below the serratus anterior muscle to achieve analgesic effects (4). Compared with traditional INB, SAPB has been reported to provide effective analgesia of the anterolateral thoracic wall (7). The serratus anterior muscle is an important muscle in the thoracic wall, located between the pectoralis major muscle and the intercostal muscles, primarily responsible for the anterior translation of the scapula, By blocking the lateral cutaneous branches of the intercostal nerves, SAPB reduces sensory transmission from the thoracic wall and contributes to postoperative pain relief (8, 9). This emerging technique has garnered increasing attention in clinical practice.

INB involves injecting anesthetic drugs into the anatomical region near the intercostal nerves to achieve paralysis of the intercostal nerves. Traditional INB can effectively relieve pain in the chest area and remains a commonly used regional analgesic technique in cardiothoracic surgery (10, 11). Additionally, INB carries risks of rib and nerve damage, especially during surgical procedures where improper manipulation or misjudgment of anatomical landmarks may lead to such complications (12).

Therefore, an increasing number of studies are comparing the analgesic effects of the SAPB and INB in cardiothoracic surgery, aiming to determine which method is more advantageous and assist clinicians in selecting appropriate analgesic strategies (13). Some preliminary studies (14, 15) suggest that the SAPB may provide comparable or potentially improved postoperative analgesia. However, whether the efficacy of the SAPB is comparable to that of INB remains a topic worthy of further exploration (16).

This systematic review and meta-analysis were designed according to a PICO framework. The Population included patients undergoing cardiothoracic surgery. The Intervention was SAPB, and the Comparator was INB. The primary Outcome was postoperative pain intensity at predefined time points, while secondary outcomes included total opioid consumption, postoperative nausea and vomiting, and length of hospital stay. This structured approach was intended to provide a focused and transparent comparison of the two analgesic strategies.

## Methods

The program was constructed in accordance with the Preferred Reporting Items for Systematic Reviews and Meta-Analyses (PRISMA) guidelines (17). This study was pre-registered with the International prospective register of systematic reviews (PROSPERO) under the registration number: CRD420251080642.

### Inclusion and exclusion criteria

Inclusion criteria:

Patients: Include adult or pediatric patients undergoing cardiac or cardiothoracic surgery (open-heart surgery, thoracoscopic surgery), without restriction based on gender, race, or region.

Intervention: studies must compare the efficacy of SAPB and INB) in postoperative pain management.

Outcomes: outcomes including pain scores (pain intensity at rest), opioid usage, and incidence of nausea and vomiting (24 h after surgery), and secondary outcomes including length of hospital stay.

Study design: study design must be RCT.

Exclusion criteria: non-original studies, such as reviews, commentaries, conference abstracts, or case reports; studies using interventions unrelated to SAPB and INB, such as other anesthetic techniques or analgesic methods; studies with incomplete data or insufficient efficacy assessment data.

### Literature search

This study conducted a literature search in PubMed, Embase, the Cochrane Library, and Web of Science. The search period spanned from the establishment of the databases to July 10, 2025. The keywords used in the search included “anterior serratus plane block”, “intercostal nerve block”, combined using Boolean operators for multidimensional combinations. The specific search strategy is detailed in Supplementary Material Table S1.

### Data extractions

Two authors (YT and PMY) independently screened the literature for inclusion by importing the literature into endnote according to the literature inclusion and exclusion criteria, the final included studies were used for data extraction using excel software and if there was a dispute about the literature screening then it would be discussed, or a third person (LSD) would be sought to adjudicate. The extracted data contained basic characteristics of the study (first author, year of publication, country), basic characteristics of the population (sample size, gender, mean age, type of surgery), intervention, and outcome. We also extracted surgical characteristics, when available, to assess baseline comparability between groups.

### Risk of bias

Risk of bias for included randomized controlled trials was assessed using the Cochrane Risk of Bias 2 (RoB2.0) tool (18). Two reviewers independently evaluated each study, and disagreements were resolved through discussion with a third reviewer. The assessment considered the five RoB 2 domains: bias arising from the randomization process; bias due to deviations from intended interventions; bias due to missing outcome data; bias in measurement of the outcome; and bias in selection of the reported result. Judgements for each domain were classified as “low risk of bias”, “some concerns”, or “high risk of bias” according to RoB 2 guidance. Domain-level judgements were based on information reported in the original publications. When reporting was unclear, the domain was rated as “some concerns”. An overall risk-of-bias judgement for each study was derived following the RoB 2 algorithm based on domain-level assessments.

### GRADE assessment

The certainty of evidence for key outcomes was evaluated using the Grading of Recommendations Assessment, Development and Evaluation (GRADE) (19) approach. Two reviewers independently assessed the quality of evidence across the domains of risk of bias, inconsistency, indirectness, imprecision, and publication bias. Evidence from randomized controlled trials was initially rated as high certainty and was downgraded when concerns were identified in any domain. Disagreements were resolved through discussion. The overall certainty of evidence for each outcome was categorized as high, moderate, low, or very low.

### Data analysis

The data analysis section utilized Stata 15.0 software (Stata Corp, College Station, TX, USA) for statistical analysis. First, heterogeneity among the included studies was assessed using the I² value or Q statistic. I² values of 0%, 25%, 50%, and 75% indicate no heterogeneity, low heterogeneity, moderate heterogeneity, and high heterogeneity, respectively. When the I² value reached or exceeded 50%, sensitivity analysis was performed to explore potential sources of heterogeneity. If heterogeneity was below 50%, a fixed-effects model was used for analysis. For continuous variables, the standardized mean difference (SMD) and its 95% confidence interval (CI) are used; for dichotomous variables, the risk ratio (RR) and its 95% confidence interval (CI) are used. Additionally, a random-effects model and Egger's test are used to assess publication bias. If the funnel plot is asymmetric, trim-and-fill methods are used for assessment to correct for potential publication bias.

## Results

### Study selection

**Figure 1:**
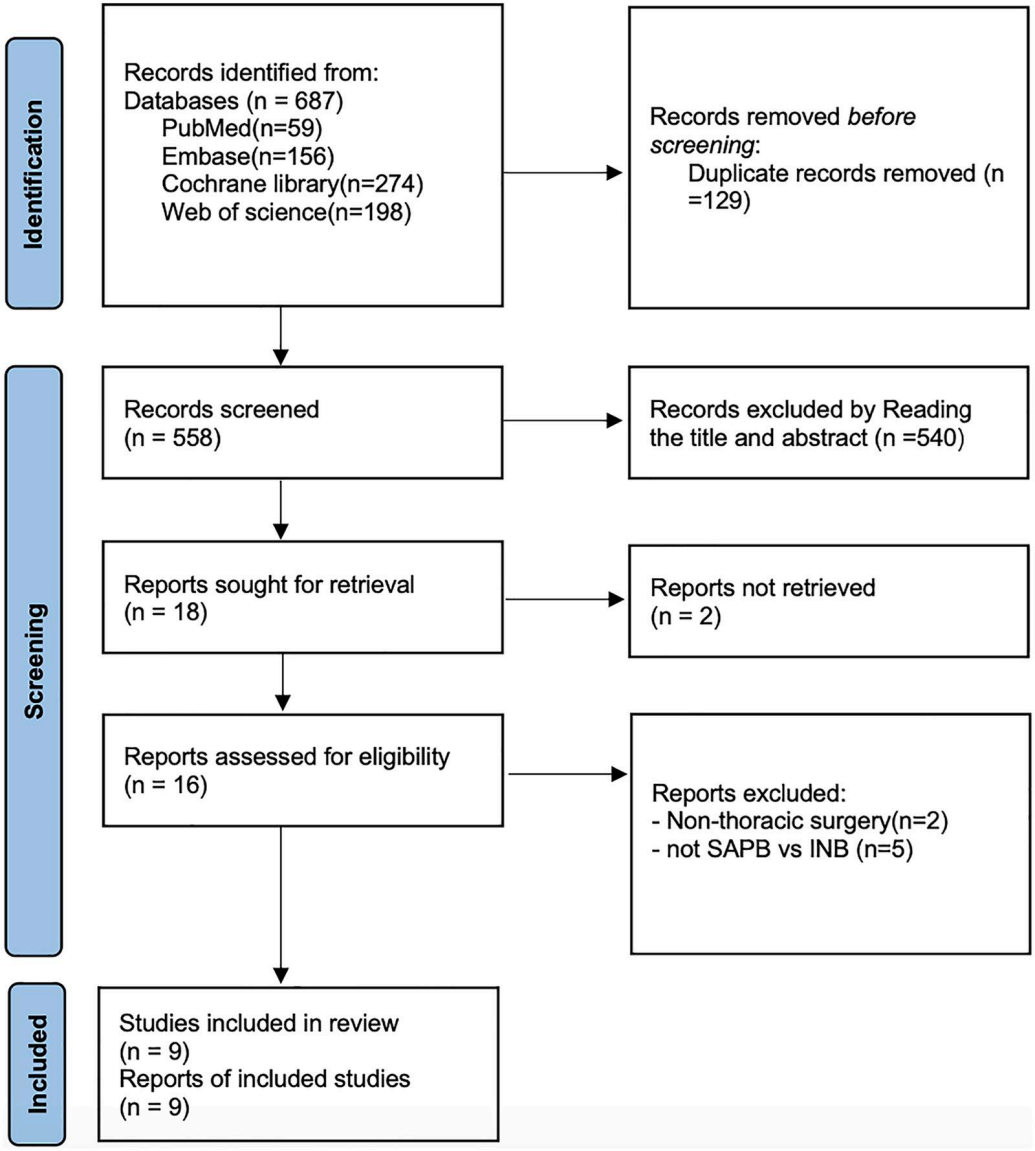
Literature search flow chart.

As shown in Figure 1, a total of 687 articles were retrieved from PubMed (*n* = 59), Embase (*n* = 156), Cochrane Library (*n* = 274), and Web of Science (*n* = 198). After removing 129 duplicates, 540 articles were excluded based on title and abstract review, and 7 articles were excluded after full-text review (Supplementary Material Table S2). Ultimately, 9 randomized controlled trials (20–28) were included.

### Basic characteristics of included studies

A total of 9 randomized controlled trials involving 606 patients were included, with 301 patients in the SAPB group and 305 in the INB group. The age range was 1.56–66.9 years, and the drugs used were ropivacaine or bupivacaine. The specific baseline characteristics are shown in Table 1.

**Table 1:** Table of basic characteristics.

Study	Year	Country	Sample size		Gender (M/F)	Mean age (years)		Type of Surgery	Type of SAPB	Measurement of pain	Intervention		Outcomes
			SAPB	INB		SAPB	INB				SAPB	INB	
Elhouty et al. (19)	2023	Egypt	71	71	NR	24.38	23.17	VATS	Superficial block	VAS	20 mL of bupivacaine 0.25% wa	20 mL of bupivacaine 0.25%	F1; F2; F3
He et al. (20)	2023	China	18	19	21/16	31.9	40.2	Thoracotomy	Deep block	NR	0.2% ropivacaine 2.5 mg.kg	0.2% ropivacaine 2.5 mg.kg	F2; F4; F5
Jin et al. (21)	2024	China	37	37	45/29	59.62	58.27	VATS	Superficial block	VAS	20 mL of 0.5% ropivacaine	20 mL of 0.5% ropivacaine	F1; F2
Kaushal et al. (22)	2019	India	36	36	39/33	1.87	1.56	Thoracotomy	Deep block	MOPS	0.2% ropivacaine 3 mg/kg	0.2% ropivacaine 3 mg/kg	F1; F2; F4
Kim et al. (23)	2021	Korea	25	25	44/6	18	18	VATS	Superficial block	NRS	20 mL of 0.375% ropivacaine	20 mL of 0.375% ropivacaine	F1; F2
Lee et al. (24)	2020	Korea	23	23	18/28	68	67	VATS	Superficial block	NRS	20 mL of 0.375% ropivacaine	20 mL of 0.375% ropivacaine	F1; F2
Lim et al. (25)	2025	Korea	30	30	29/31	65.5	66.9	VATS	Superficial block	VAS	35 mL of 0.25% ropivacaine	35 mL of 0.25% ropivacaine	F1; F5
Magoon et al. (26)	2020	India	30	30	33/27	24.86	24.77	thoracotomy	Deep block	VAS	30 mL of 0.25% ropivacaine	30 mL of 0.25% ropivacaine	F1; F2; F5
Pai et al. (27)	2022	USA	35	30	28/36	53.3	64.3	VATS	Deep block	VAS	20 mL of 0.375% ropivacaine	20 mL of 0.375% ropivacaine	F1; F2; F3; F5

SAPB, serratus anterior plane block; INB, intercostal nerve blocks; M/F, male/female; F1, pian scores; F2, opioid consumption; F3, 1st Request of analgesia (minutes); F4, Nausea/vomiting; F5, Length of hospital.

### Risk of bias results

Risk of bias was assessed using the Cochrane Risk of Bias tool (RoB 2). Random sequence generation was adequate in all studies, but allocation concealment was insufficiently reported in several trials, resulting in some concerns for this element. All studies were low risk for deviations from intended interventions and missing outcome data. Some concerns were noted in outcome measurement due to unclear assessor blinding, while selective reporting was low risk across studies. Most trials showed some concerns in the other bias domain, and none were judged high risk. Detailed assessments are presented in Supplementary Figures S1, S2.

Certainty of evidence was evaluated using the GRADE approach (Supplementary Material Table S3). Due to concerns regarding risk of bias and inconsistency, the overall certainty of evidence for these outcomes was rated as low, indicating that the findings should be interpreted with caution.

### Meta analysis-results

#### 6 h pain scores

**Figure 2:**
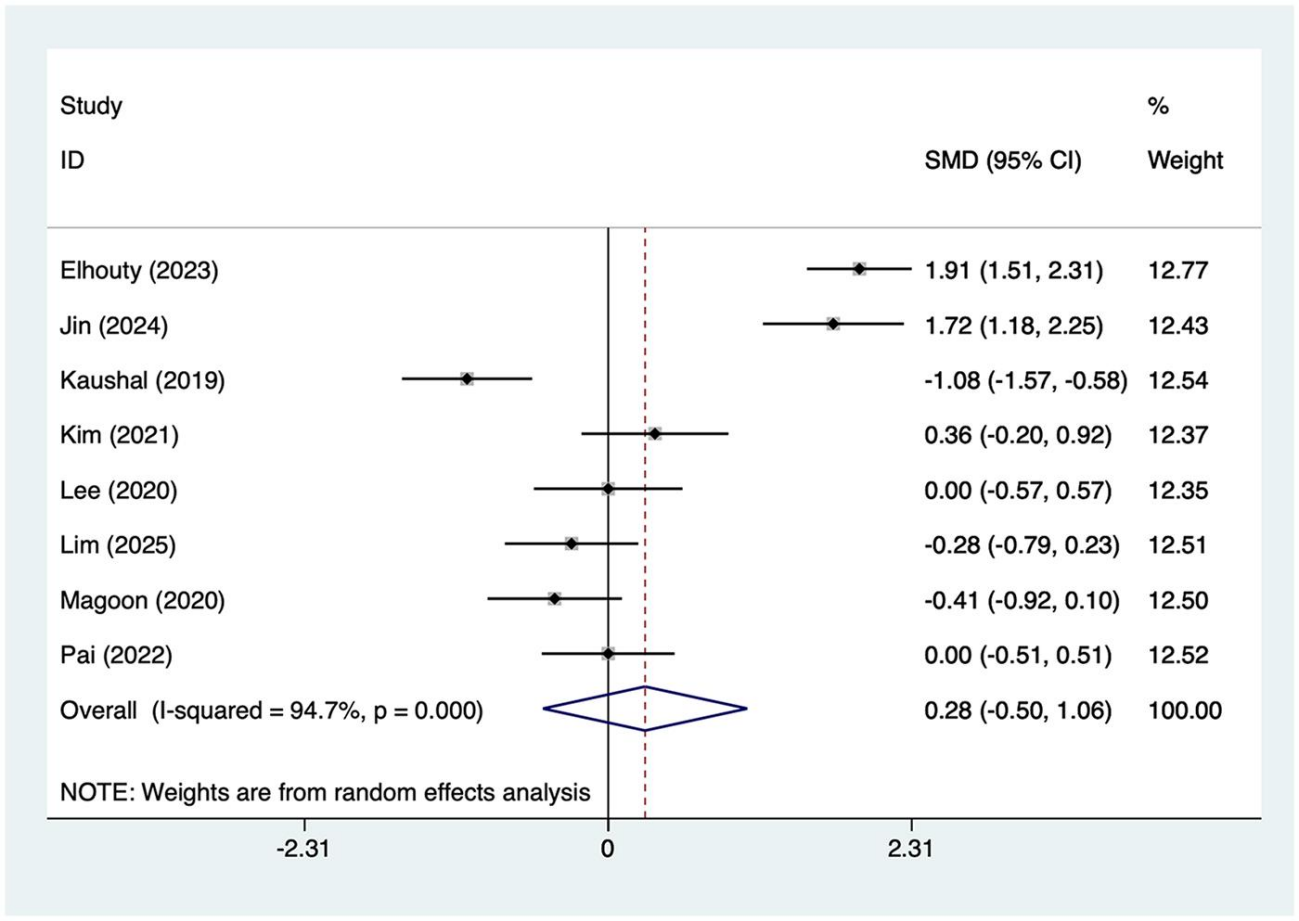
Forest plot of 6 h pain score.

8 articles mentioned the 6 h pain score, with a heterogeneity test (*I*^2^ = 94.7%, *P* = 0.001). Analysis was conducted using a random-effects model. The results (Figure 2) indicated that no differences in 6-h pain scores between the SAPB group and the INB group [SMD = 0.28, 95% CI (−0.50, 1.06)]. Due to the high heterogeneity, a sensitivity analysis was conducted by sequentially excluding individual studies. The results (Supplementary Material Figure S3) suggested that this indicator was not influenced by the results of any single study. This study conducted subgroup analyses based on Type of Surgery, Type of SAPB, and Measurement of pain. The results (Table 2) indicate that for thoracotomy procedures, SAPB demonstrated superior efficacy compared to INB in 6 h pain scores [SMD = −0.74, 95% CI (−1.4, −0.09)]. No significant differences were observed in the remaining subgroups.

**Table 2:** Results of subgroup meta-analysis.

Outcomes	Group	Subgroup	No of study	Heterogeneity (*I*^2^%)	SMD (95% CI)	*P*
6 h-pain scores	Type of Surgery	VATS	6	93.6	0.62 (−0.19, 1.44)	0.12
		Thoracotomy	2	70.3	−0.74 (−1.4, −0.09)	0.004
	Type of SAPB	Superficial block	5	94.1	0.75 (−0.18, 1.68)	0.42
		Deep block	3	77.9	−0.50 (−1.12, 0.12)	0.19
	Measurement of pain	VAS	5	95.5	0.59 (−0.44, 1.62)	0.27
		MOPS	1	NA	−1.08 (−1.57, −0.58)	0.003
		NRS	2	0	0.18 (−0.22, 0.58)	0.54
12 h-pain scores	Type of Surgery	VATS	5	95.5	0.03 (−1.03, 1.08)	0.48
		Thoracotomy	2	87.2	−2.14 (−3.36, −0.91)	0.001
	Type of SAPB	Superficial block	5	95.5	0.03 (−1.03, 1.08)	0.48
		Deep block	2	87.2	−2.14 (−3.36, −0.91)	0.001
	Measurement of pain	VAS	4	97.6	−0.34 (−1.91, 1.23)	0.57
		MOPS	1	NA	−2.77 (−3.42, −2.11)	0.01
		NRS	2	0	−0.03 (−0.43, 0.37)	0.93
24 h-pain scores	Type of SAPB	Superficial block	4	84.2	−0.30 (−0.80, 0.29)	0.42
		Deep block	1	NA	0.87 (0.34, 1.40)	0.02
	Measurement of pain	VAS	4	90.5	−0.09 (−0.83, 0.65)	0.93
		NRS	1	NA	0.00 (−0.58, 0.58)	0.61
Total opioid consumption	Type of Surgery	VATS	5	97.4	−2.38 (−4.08, −0.67)	0.001
		Thoracotomy	3	96.5	−1.38 (−3.37, 0.60)	0.17
	Type of SAPB	Superficial block	4	98.1	−2.61 (−4.86, −0.36)	0.023
		Deep block	4	95.2	−1.42 (−2.83, −0.02)	0.047

#### 12 h pain scores

**Figure 3:**
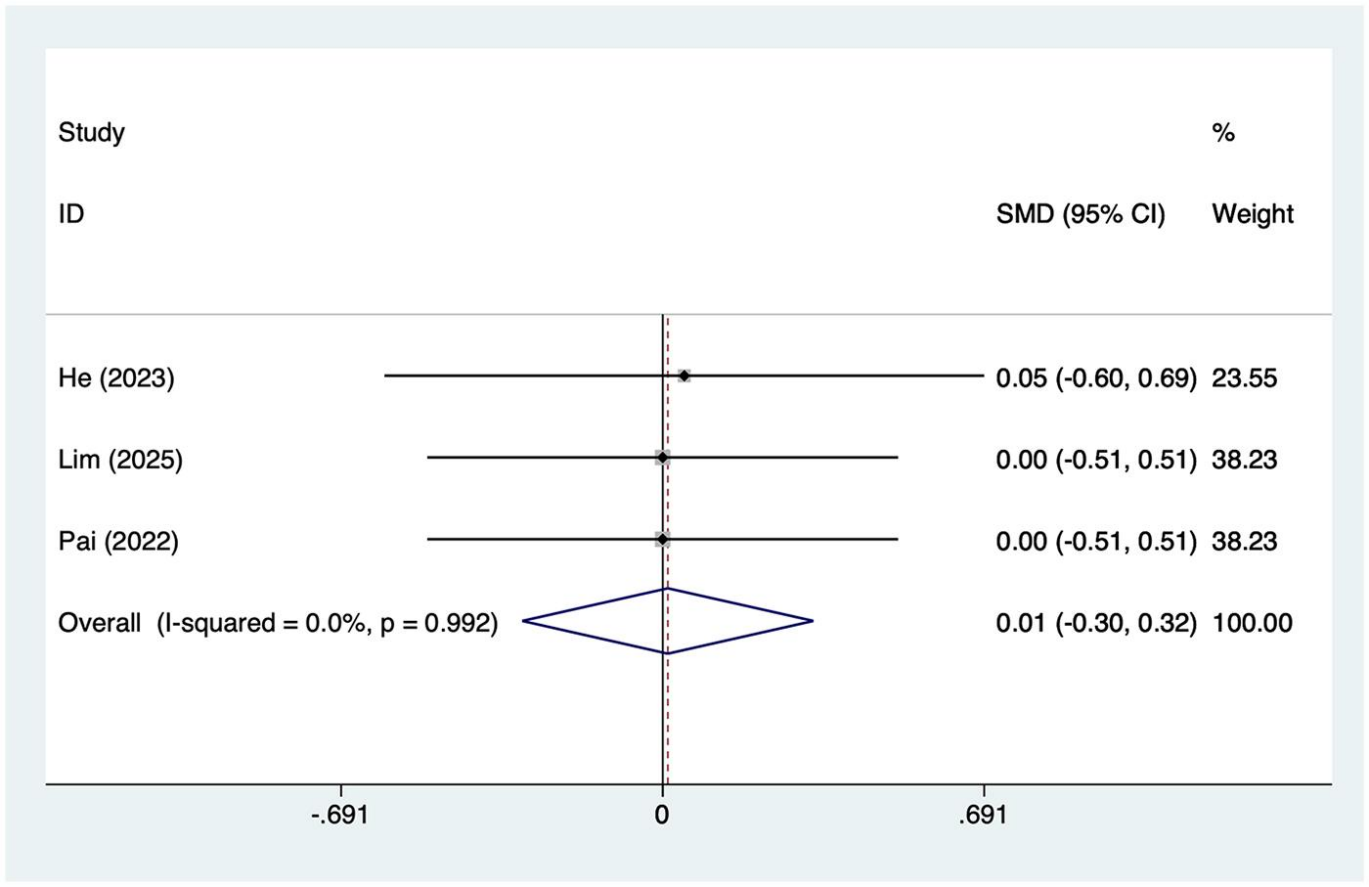
Forest plot of 12 h pain score.

7 articles mentioned the 12 h pain score, with a heterogeneity test (*I*^2^ = 96.9%, *P* = 0.001). Analysis was conducted using a random-effects model. The results (Figure 3) indicated that no differences in 12 h pain scores between the SAPB group and the INB group [SMD = −0.59, 95% CI (−1.71, 0.53)]. Due to the high heterogeneity, a sensitivity analysis was conducted by sequentially excluding individual studies. The results (Supplementary Material Figure S4) suggested that this indicator was not influenced by the results of any single study. This study conducted subgroup analyses based on Type of Surgery, Type of SAPB, and Measurement of pain. The results (Table 2) indicate that for thoracotomy procedures, SAPB demonstrated superior efficacy compared to INB in 12 h pain scores [SMD = −2.14, 95% CI (−3.36, −0.91)], for Deep block SAPB, the 12 h pain score was significantly better than INB [SMD = −2.14, 95% CI (−3.36, −0.91)], with no significant differences observed in the remaining subgroups.

#### 24 h pain scores

**Figure 4:**
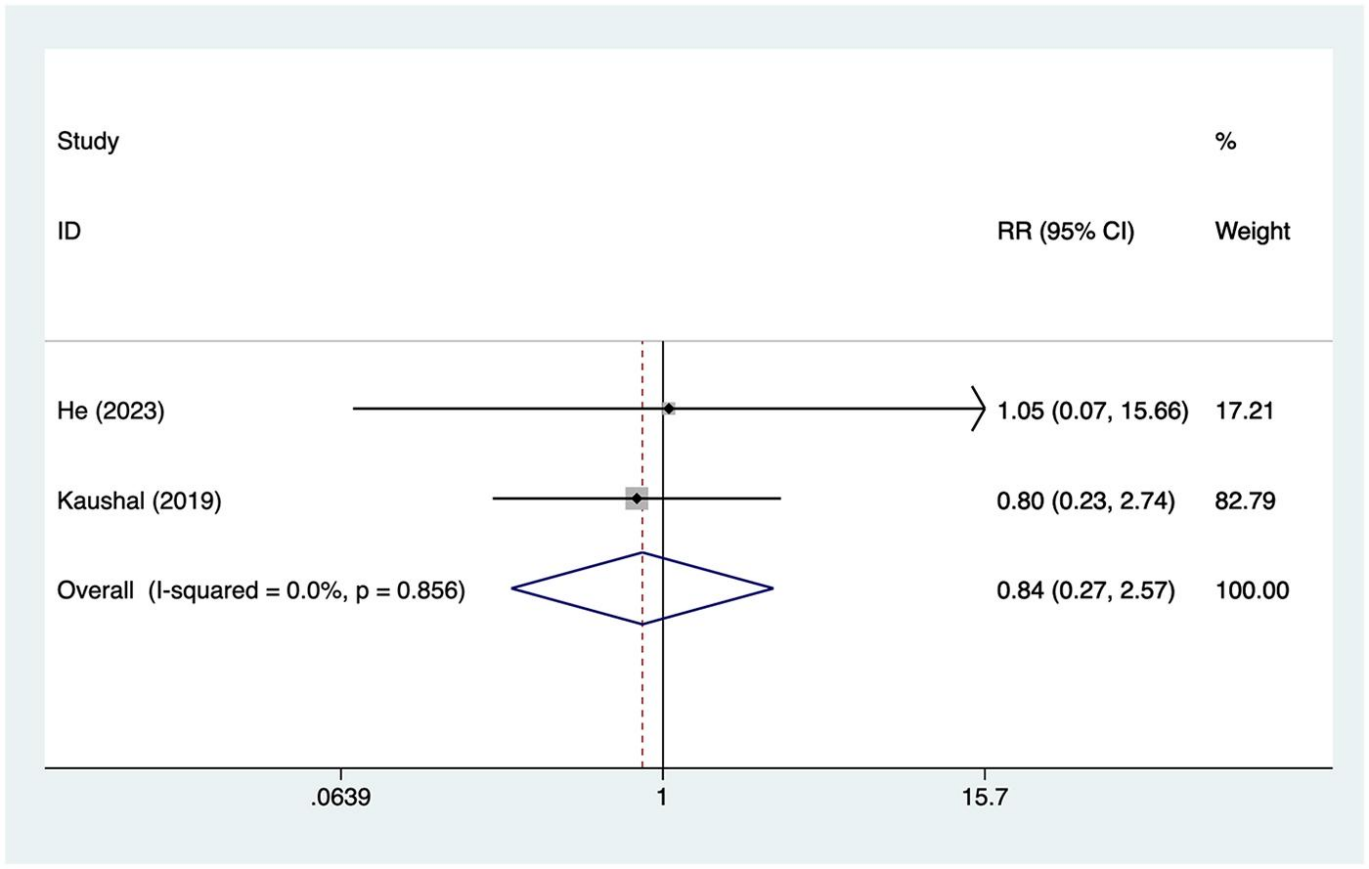
Forest plot of 24 h pain score.

5 articles mentioned the 24 h pain score, with a heterogeneity test (*I*^2^ = 87.3%, *P* = 0.001). Analysis was conducted using a random-effects model. The results (Figure 4) indicated that no differences in 24 h pain scores between the SAPB group and the INB group [SMD = −0.07, 95% CI (−0.67, 0.52)]. Due to the high heterogeneity, a sensitivity analysis was conducted by sequentially excluding individual studies. The results (Supplementary Material Figure S5) suggested that this indicator was not influenced by the results of any single study. This study conducted subgroup analyses based on Type of SAPB, and Measurement of pain (Table 2), with no significant differences observed in all subgroups.

#### Total opioid consumption

**Figure 5:**
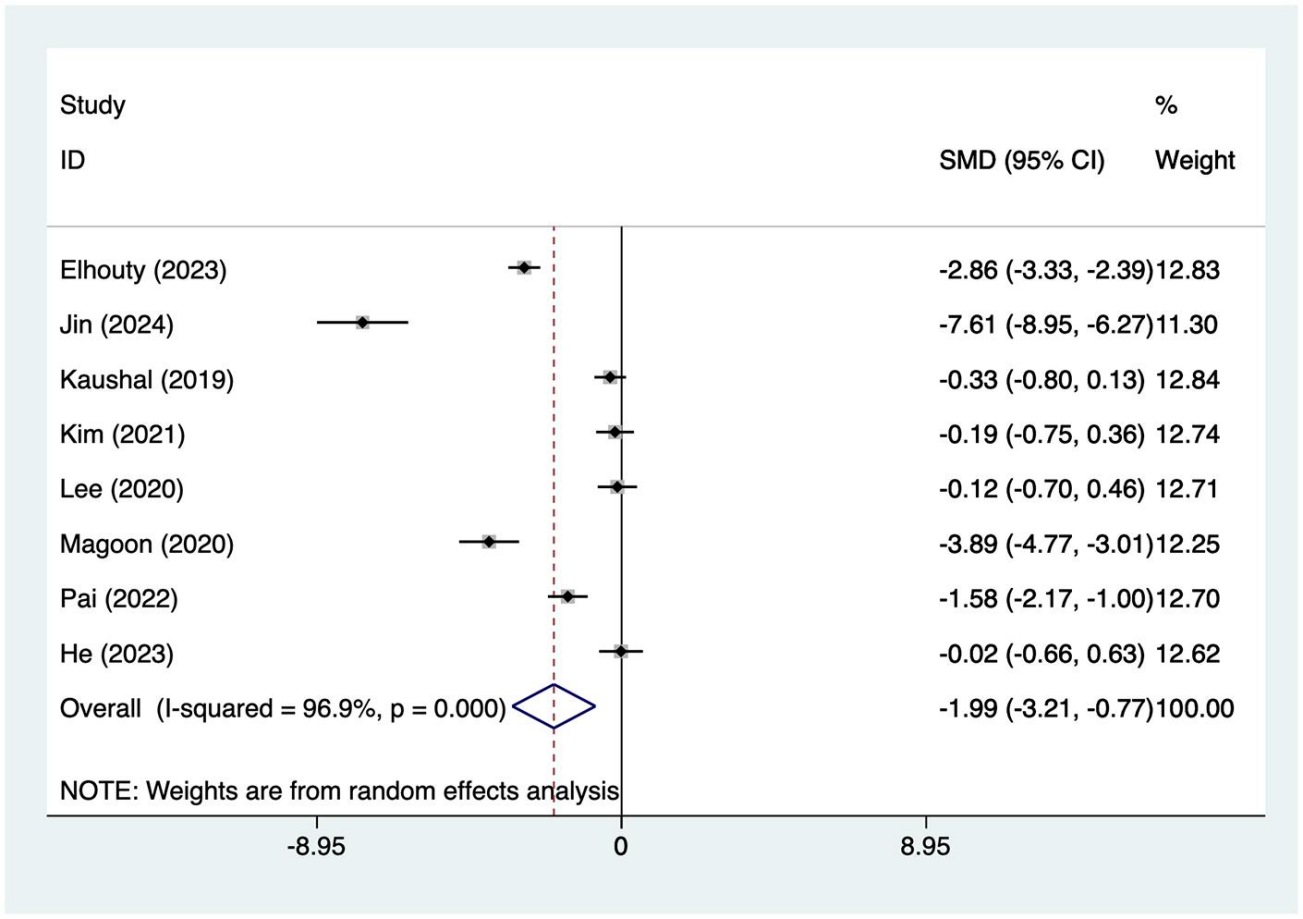
Forest plot of total opioid consumption.

Eight studies reported total opioid consumption. Substantial heterogeneity was observed (*I*^2^ = 96.9%, *P* = 0.001); therefore, a random-effects model was applied. Because different opioid types and dosing regimens were used across studies, SMD were calculated to pool outcomes measured on different scales. The results (Figure 5) indicated that the total opioid consumption was lower in the SAPB group than in the INB group [SMD = −1.99, 95% CI (−3.21, −0.77)]. Due to the high heterogeneity, a sensitivity analysis was conducted by sequentially excluding individual studies. The results (Supplementary Material Figure S6) suggested that this indicator was not influenced by the results of any single study. Subgroup analysis was performed according to Type of Surgery and Type of SAPB, and the results (Table 2) suggested that for VATS [SMD = −2.38 (−4.08, −0.67)], and for Superficial block [SMD = −2.61 (−4.86, −0.36)], Deep block [SMD = −1.42 (−2.83, −0.02)], SAPB had lower total opioid consumption than INB.

#### Nausea/vomiting

**Figure 6:**
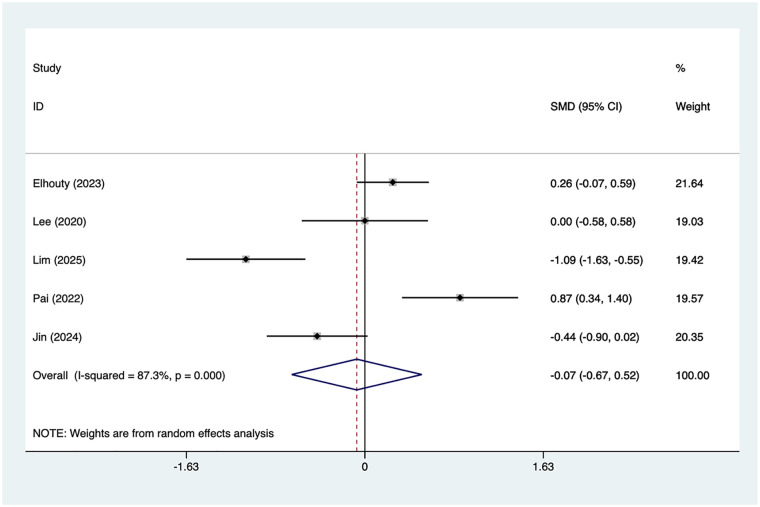
Forest plot of nausea/vomiting.

2 articles mentioned the nausea/vomiting, with a heterogeneity test (*I*^2^ = 0%, *P* = 0.856). Analysis was conducted using a fixed-effects model. The results (Figure 6) indicated that there was no difference in the incidence of nausea and vomiting between the SAPB group and the INB group [RR = 0.84, 95% CI (0.27, 2.57)].

#### Length of hospital stay

**Figure 7:**
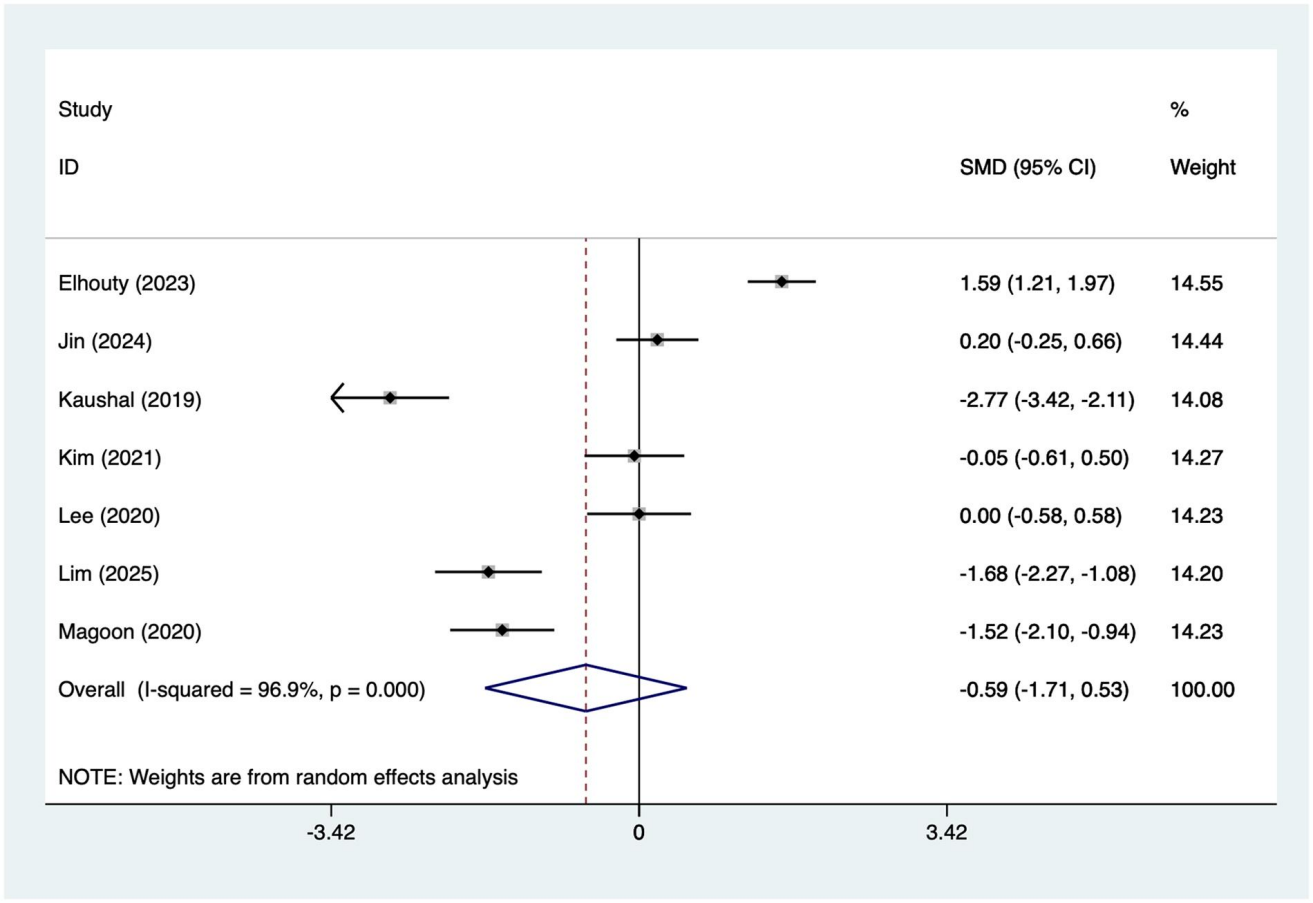
Forest plot of length of hospital stay.

3 articles mentioned the length of hospital stay, with a heterogeneity test (*I*^2^ = 0%, *P* = 0.992). Analysis was conducted using a fixed-effects model. The results (Figure 7) indicated that there was no difference in the length of hospital stay between the SAPB group and the INB group [SMD = 0.01, 95% CI (−0.30, 0.32)].

#### Publication bias

This study used funnel plots and the Egger test to assess publication bias. The analysis results (Supplementary Materials Figures S7–S10) indicate that the funnel plots are relatively symmetrical, although visual inspection of funnel plots is inherently subjective, particularly when the number of included studies is small. The Egger test showed no significant interaction for 6 h pain scores (*P* = 0.191), 12 h pain scores (*P* = 0.113), 24 h pain scores (*P* = 0.715), and total opioid consumption (*P* = 0.133); all *P* values were greater than 0.05. However, given the relatively limited number of studies included for these outcomes, the statistical power of both funnel plot asymmetry assessment and Egger's regression test is constrained. It is generally recognized that when fewer than 10 studies are available, asymmetry tests may lack sufficient sensitivity to detect small-study effects. Therefore, the absence of statistically significant findings should not be interpreted as definitive evidence of no publication bias, and potential small-study effects cannot be completely excluded.

For nausea/vomiting and length of hospital stay, publication bias was not assessed due to limited data. Given the small number of studies reporting these outcomes, formal evaluation of publication bias would not have been methodologically robust.

## Discussion

This meta-analysis evaluated the analgesic efficacy and other clinical outcomes of the SAPB vs. the INB in cardiothoracic surgery. The analysis suggested no significant difference between SAPB and INB in pain scores at 6, 12, and 24 h. However, the SAPB group was associated with lower pooled estimates of total opioid consumption compared to the INB group. Furthermore, in specific subgroup analyses, SAPB appeared to show potential advantages for certain types of surgeries. The more pronounced differences observed in the thoracotomy subgroup may be attributable to the higher baseline pain intensity and greater surgical trauma associated with open procedures, although the substantial heterogeneity and limited subgroup sample sizes mean these findings should be interpreted cautiously rather than as confirmatory evidence.

Pain scores were the primary outcomes in most included studies and were assessed using subjective scales such as the VAS or NRS. As these outcomes rely on patient self-reporting, they are inherently susceptible to performance and detection bias. In several trials, blinding of participants and healthcare providers was either not feasible or not clearly described, which may have influenced patients' perception of pain as well as clinicians' postoperative management. Lack of adequate blinding may lead to expectation effects, particularly when comparing two different regional anesthesia techniques. Furthermore, outcome assessors were not consistently blinded across studies. Given that pain assessment is subjective and may be influenced by patient–clinician interactions, incomplete blinding could have introduced detection bias. Therefore, although most studies were randomized, the potential influence of performance and detection bias together with variability in outcome reporting may contribute to uncertainty in pooled estimates and should be considered when interpreting the results.

First, regarding pain scores at 6, 12, and 24 h, despite all relevant studies employing random-effects models and sensitivity analyses ruling out interference from individual studies, results still showed minimal differences in pain scores between the two groups at these time points. Overall, these findings suggest broadly comparable short-term analgesic effects between SAPB and INB; however, the substantial heterogeneity observed in several analyses' limits confidence in precise effect estimation and warrants cautious interpretation. Specifically, for the 6 h pain score, although high heterogeneity (*I*^2^ = 94.7%) may have affected the stability of the results, sensitivity analyses did not alter this conclusion. Subgroup analysis indicated that SAPB outperformed INB in 6 h pain scores during thoracotomy. Nevertheless, this subgroup finding should be interpreted cautiously given the limited number of studies within subgroups and the exploratory nature of these analyses. However, no significant difference was observed for other surgical types. Comparisons of 12 h pain scores yielded results like those at 6 h. The SAPB group appeared to show possible advantages in certain subgroups, particularly in the open-chest surgery cohort. However, no significant difference in analgesic effect was observed between SAPB and INB in subgroups assessed by VAS and NRS. These inconsistencies, together with non-standardised outcome reporting, prevent firm conclusions regarding differential efficacy, particularly in specific surgical types. However, these subgroup differences were not consistently observed across all classification methods (VAS vs. NRS) and therefore do not provide definitive evidence of superiority in specific surgical settings. For 24 h pain scores, results showed no significant difference between SAPB and INB. Notably, in the deep block group, SAPB was significantly less effective. This isolated finding may indicate that SAPB's efficacy in relieving persistent pain may be limited in certain scenarios; however, this interpretation is constrained by limited data and residual heterogeneity (29). The lack of significant difference in analgesic efficacy between SAPB and INB may be partly explained by similarities in their targeted neural pathways; however, the present meta-analysis was not designed to directly evaluate mechanistic differences (30). Although they act through distinct anatomical approaches—SAPB relieves pain by blocking intercostal and intrathoracic nerves within the fascial plane, while INB directly injects anesthetic into the intercostal spaces—both methods ultimately aim to reduce postoperative thoracic pain by inhibiting nociceptive transmission (31). Pain transmission in the chest involves multiple neural pathways, particularly the intercostal and intrathoracic nerves, which exhibit anatomical overlap, such overlap may contribute to comparable clinical effects, but this hypothesis requires confirmation in dedicated mechanistic studies. Additionally, the diffusion range of anesthetic agents may contribute to the minimal observed differences (32). Although SAPB covers a larger area of the serratus anterior muscle, insufficient diffusion of the anesthetic agent may prevent complete pain signal blockade, resulting in suboptimal outcomes (33). Similarly, after injection into the intercostal space, the INB's effect may be limited, failing to significantly improve pain perception in some patients. Collectively, these factors contribute to the lack of significant difference in analgesic efficacy between SAPB and INB (34). However, variability in local anesthetic type, concentration, and volume across studies limits definitive conclusions regarding diffusion-related effects.

SAPB was associated with a possible advantage over INB in reducing the overall use of opioid medications, particularly in patient populations requiring lower doses. However, high heterogeneity (*I*^2^ = 96.8%) considerable between-study variability. SAPB may effectively reduce local pain perception by anesthetizing the serratus anterior muscle and its associated nerves (35). In contrast, the anesthetic effect of INB primarily targets the intercostal nerves. While intercostal nerve block is widely used for chest wall pain management, it may lead to higher postoperative demand for analgesic medications among patients (36). Therefore, SAPB can play a role in reducing opioid use, particularly in the early postoperative period, as the alleviation of pain perception may reduce patients' reliance on excessive opioid medications. However, SAPB cannot completely replace INB, as its anesthetic effect is relatively limited and may not effectively cover all pain conduction pathways (7). INB directly acts on intercostal nerves to broadly block pain signal transmission, but this may also lead to patients requiring additional opioid medications to supplement analgesic effects (37). Results indicate that in more localized pain control, SAPB may reduce opioid use, while INB, due to its broader coverage of neural regions, relies more heavily on opioid medications in postoperative pain management (38). Although a large, standardized effect size was observed (SMD = −1.99), this value reflects a standardized difference rather than an absolute reduction in opioid dose. Because opioid types, administration routes, and dosing regimens varied considerably across studies, and uniform conversion to morphine milligram equivalents was not consistently feasible, the magnitude of the SMD may be influenced by between-study variability. Therefore, while the findings suggest that SAPB may reduce opioid requirements compared with INB, the clinical significance in terms of absolute dose reduction remains uncertain. Future trials should report opioid consumption in standardized morphine milligram equivalents to improve interpretability and comparability.

In the comparison of nausea/vomiting incidence and hospital stay duration, there was no significant difference between the SAPB group and the INB group. Although SAPB demonstrated superior efficacy in reducing opioid consumption, it did not appear to significantly improve postoperative gastrointestinal function (39). Nausea and vomiting result from the interplay of multiple factors, including not only the use of analgesic medications but also the patient's overall health status, anesthetic techniques, and surgical procedures, all of which may influence postoperative gastrointestinal responses (40). Therefore, future studies should further explore the applicability of SAPB across different surgical procedures and patient populations, particularly in patients sensitive to gastrointestinal responses, to determine whether optimizing analgesic regimens can further reduce postoperative discomfort (41). Additionally, there was no significant difference in length of hospital stay between the two groups, suggesting that while SAPB may offer advantages in pain control, its impact on hospital stay may be influenced by other factors (42). Hospital stay is typically determined by multiple factors such as postoperative complications and recovery progress. Therefore, while pain management is an important factor influencing recovery, it may not be sufficient to independently alter hospital stay. Current evidence does not support definitive conclusions regarding its impact on broader recovery outcomes.

It is important to acknowledge that postoperative pain following cardiothoracic surgery is influenced not only by the choice of regional anesthesia technique but also by surgical factors, including the surgical approach, extent of tissue trauma, and drainage management strategies (number and placement of chest tubes). Unfortunately, detailed information regarding drainage techniques was inconsistently reported across the included studies, precluding stratified or adjusted analyses based on these variables. Therefore, it cannot be excluded that differences in drainage practices may have partially influenced the observed analgesic effects, particularly in patients undergoing thoracotomy, where postoperative pain is generally more severe.

Regarding the absence of significant differences in the VATS subgroup, it is possible that the relatively standardized and minimally invasive nature of VATS procedures, potentially including more uniform drainage strategies, may have reduced variability in postoperative pain. However, due to limited reporting of drainage-related details, this hypothesis could not be formally evaluated in the present analysis. Future randomized trials should provide standardized reporting of surgical and drainage-related variables to allow more precise assessment of analgesic efficacy.

Although this study did not identify significant publication bias, funnel plots and Egger's test indicated low power for detecting bias. It should be noted, however, that the statistical power of existing bias assessment methods is limited due to the relatively small number of studies included in each analysis. Therefore, we recommend exercising appropriate caution when interpreting these results. Future studies could enhance the statistical power of publication bias tests by increasing sample sizes, thereby further validating our conclusions.

Substantial heterogeneity was observed across several pooled outcomes. To account for between-study variability, random-effects models were applied in all meta-analyses. The observed heterogeneity may be attributable to differences in surgical approach (VATS vs. thoracotomy), extent of resection, block technique (superficial vs. deep SAPB), local anesthetic regimens, and perioperative analgesic protocols across studies. Subgroup analyses were conducted to explore potential sources of heterogeneity, and some reduction in heterogeneity was observed within more clinically homogeneous subgroups. In addition, sensitivity analyses were performed to assess the robustness of the findings. Nevertheless, given the magnitude of heterogeneity in certain outcomes, the pooled estimates should be interpreted with caution. The results should be considered as providing an overall trend rather than definitive evidence of superiority of one technique over the other. Subgroup analyses were performed to explore potential sources of heterogeneity; however, several subgroups were based on a small number of studies, limiting statistical power and increasing the risk of spurious findings. These analyses should therefore be regarded as hypothesis-generating. Future large-scale randomized controlled trials are needed to confirm whether surgical approach or block depth truly modifies the comparative effectiveness of SAPB and INB.

## Clinical significance

The results of this study indicate that SAPB and INB demonstrate comparable analgesic efficacy in cardiothoracic surgery, with no significant differences observed in pain scores at 6, 12, and 24 h. This finding holds significant clinical importance, as the relative equivalence of these two anesthetic techniques in pain management provides greater flexibility for clinicians. Specifically, physicians can flexibly select the anesthetic method based on specific circumstances, particularly in addressing the diverse needs of different surgical procedures and patients. Furthermore, SAPB demonstrates certain advantages in reducing opioid consumption, effectively lowering postoperative opioid usage while minimizing opioid-related side effects and abuse risks. This holds significant clinical value for improving postoperative recovery quality and reducing medication dependency. Therefore, although both anesthesia methods show minimal differences in pain control, SAPB may offer additional clinical benefits in reducing drug consumption.

## Strengths and limitations

Strengths: This study employed rigorous meta-analysis methods to comprehensively evaluate the analgesic efficacy and other clinical outcomes of SAPB vs. INB in cardiothoracic surgery, yielding high-level evidence. Through multiple subgroup analyses, it revealed the impact of different surgical types, anesthetic techniques, and pain assessment criteria on analgesic outcomes, enhancing the reliability and applicability of its conclusions. Furthermore, sensitivity analyses further excluded interference from individual studies, ensuring robust findings. Concurrently, the study addressed the clinical value of reducing opioid consumption, highlighting potential advantages of SAPB over INB. This aids clinicians in considering opioid reduction during anesthesia selection, thereby improving postoperative recovery.

Limitations: Despite providing a comprehensive comparative analysis, this study has certain limitations. First, the number of included studies was relatively small, and significant heterogeneity existed, particularly regarding surgical types, anesthetic techniques, and pain assessment criteria. This may have impacted the generalizability and accuracy of the findings. Second, although sensitivity analyses did not reveal significant bias, differences in patient populations, postoperative care protocols, and assessment standards across studies could still influence the results. Finally, this study primarily focused on short-term analgesic efficacy and opioid consumption, lacking an assessment of long-term outcomes and patient prognosis between the two anesthesia methods. Future research should further explore differences in these aspects. The number of studies for each outcome was insufficient to support robust meta-regression models in our analyses. Therefore, subgroup analyses were performed to explore potential sources of heterogeneity. However, the lack of meta-regression is a limitation, and future studies with larger sample sizes will be able to more fully explore sources of heterogeneity.

## Conclusion

This meta-analysis found no statistically significant differences between SAPB and INB in pain scores at 6, 12, and 24 h. Although some subgroup analyses suggested potential differences in specific surgical settings, these findings are exploratory and should be interpreted with caution. SAPB was associated with lower reported opioid consumption; however, substantial heterogeneity and variability in opioid measurement limit the certainty and clinical relevance of this observation. No significant differences were observed in nausea/vomiting or length of hospital stay. Overall, current evidence suggests broadly comparable analgesic efficacy between SAPB and INB, but definitive comparative conclusions are limited by methodological heterogeneity and sample size constraints. Further high-quality randomized controlled trials are warranted.

## Data Availability

The original contributions presented in the study are included in the article/Supplementary Material, further inquiries can be directed to the corresponding author.
