# Supplementary material for: Efficacy of anterior serratus plane block and intercostal nerve block in cardiothoracic surgery: a meta-analysis
Source: Front Surg. 2026 Mar 11;13:1749519. doi: 10.3389/fsurg.2026.1749519 (PMC13014099; doi:10.3389/fsurg.2026.1749519)
Supplement: Supplementary file 1 [file Supplementaryfile1.docx]

Table S1 search strategy

| PubMed | ((((serratus anterior plane block[Title/Abstract]) OR (serratus plane block[Title/Abstract])) OR (serratus anterior block[Title/Abstract])) OR (SAPB[Title/Abstract])) AND (((intercostal nerve block[Title/Abstract]) OR (intercostal block[Title/Abstract])) OR (INB[Title/Abstract])) |
| --- | --- |
| Embase | \| #12 \| #6 AND #11 \| \| --- \| --- \| \| #11 \| #7 OR #8 OR #9 OR #10 \| \| #10 \| 'inb':ab,ti \| \| #9 \| 'intercostal block':ab,ti \| \| #8 \| 'intercostal nerve block':ab,ti \| \| #7 \| 'intercostal nerve block'/exp \| \| #6 \| #1 OR #2 OR #3 OR #4 OR #5 \| \| #5 \| 'sapb':ab,ti \| \| #4 \| 'serratus anterior block':ab,ti \| \| #3 \| 'serratus plane block':ab,ti \| \| #2 \| 'serratus anterior plane block':ab,ti \| \| #1 \| 'serratus anterior plane block'/exp \| |
| Cochrane library | #1 MeSH descriptor: [] explode all trees 0  #2 (serratus anterior plane block):ti,ab,kw OR (serratus plane block):ti,ab,kw OR (serratus anterior block):ti,ab,kw OR (SAPB):ti,ab,kw 972  #3 #1or#2 972  #4 MeSH descriptor: [] explode all trees 0  #5 (intercostal nerve block):ti,ab,kw OR (intercostal block):ti,ab,kw OR (INB):ti,ab,kw 1399  #6 #4or#5 1399  #7 #3and#6 275 |
| Web of science | \| 1 \| TS=(serratus anterior plane block) OR TS=(serratus plane block) OR TS=(serratus anterior block) OR TS=(SAPB) \| \| --- \| --- \| \| 2 \| TS=(intercostal nerve block) OR TS=(intercostal block) OR TS=(INB) \| \| 3 \| #1 AND #2 \| |

Table s2 Details of the study were excluded

| Study | Doi | reasons for removal |
| --- | --- | --- |
| Jiang 2021 | 10.1111/ijcp.14539 | Non-thoracic surgery |
| Zhang 2022 | 10.1155/2022/6924489 | not SAPB vs INB |
| Zengin 2025 | 10.1186/s12871-025-03000-6 | Non-thoracic surgery |
| Yu 2025 | 10.1186/s12871-025-03049-3 | Non-thoracic surgery |
| Wang 2024 | 10.2147/jpr.S484092 | not SAPB vs INB |
| Ghaffar 2025 | 10.21608/asja.2024.280185.1088 | not SAPB vs INB |
| Chaudhary2020 | 10.1016/j.athoracsur.2020.03.117 | not SAPB vs INB |

Table S3 grade results

| Outcomes | grade |
| --- | --- |
| **6h pain scores** | low |
| **12h pain scores** | low |
| **24h pain scores** | low |


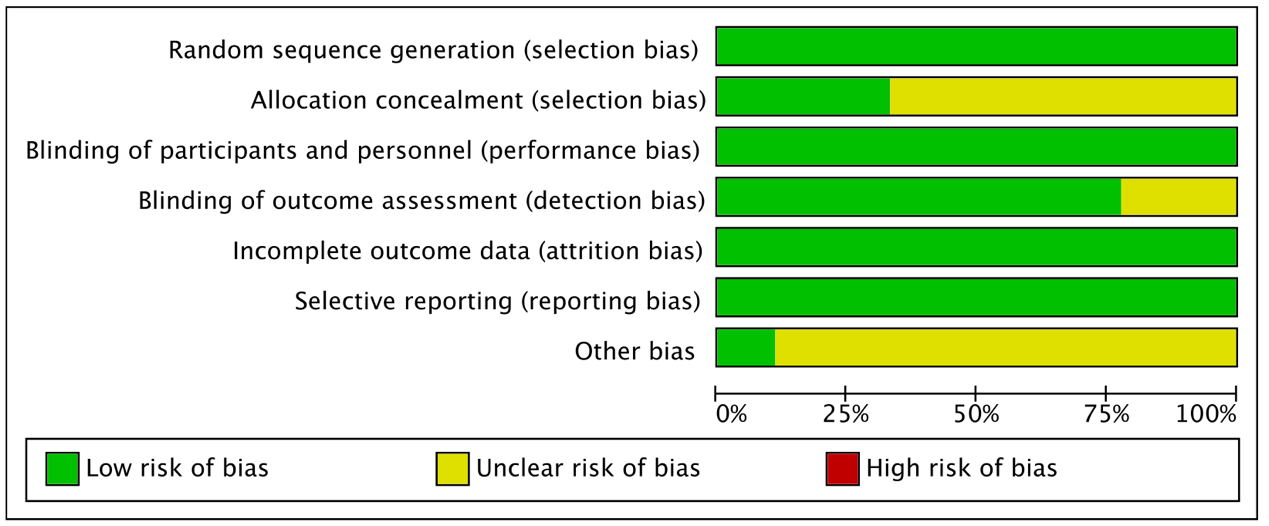


Figure S1 risk of bias graph


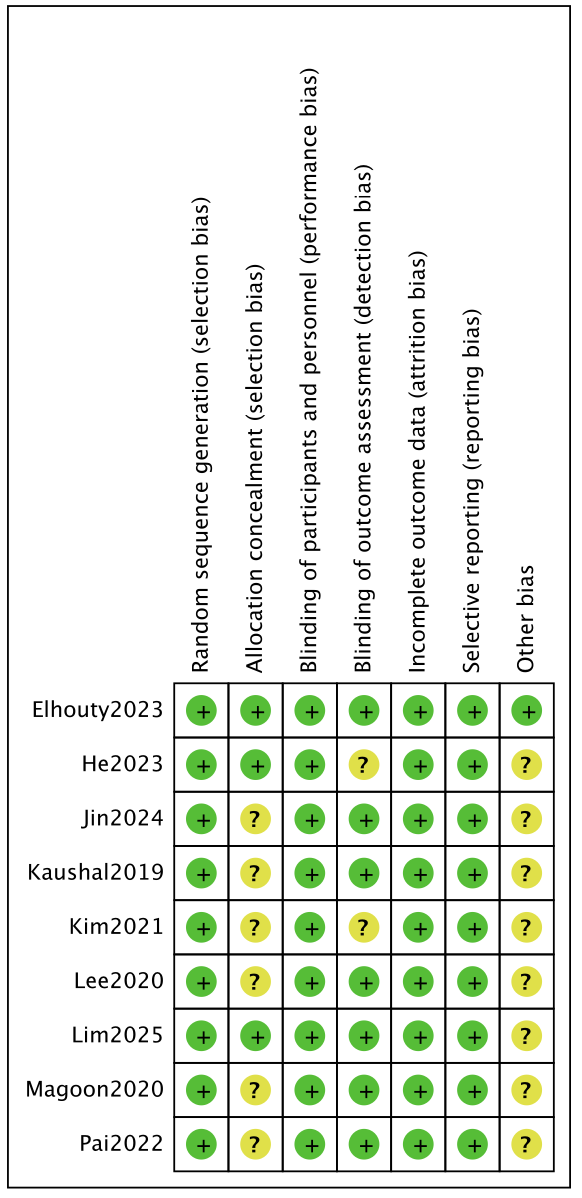


Figure S2 risk of bias summary


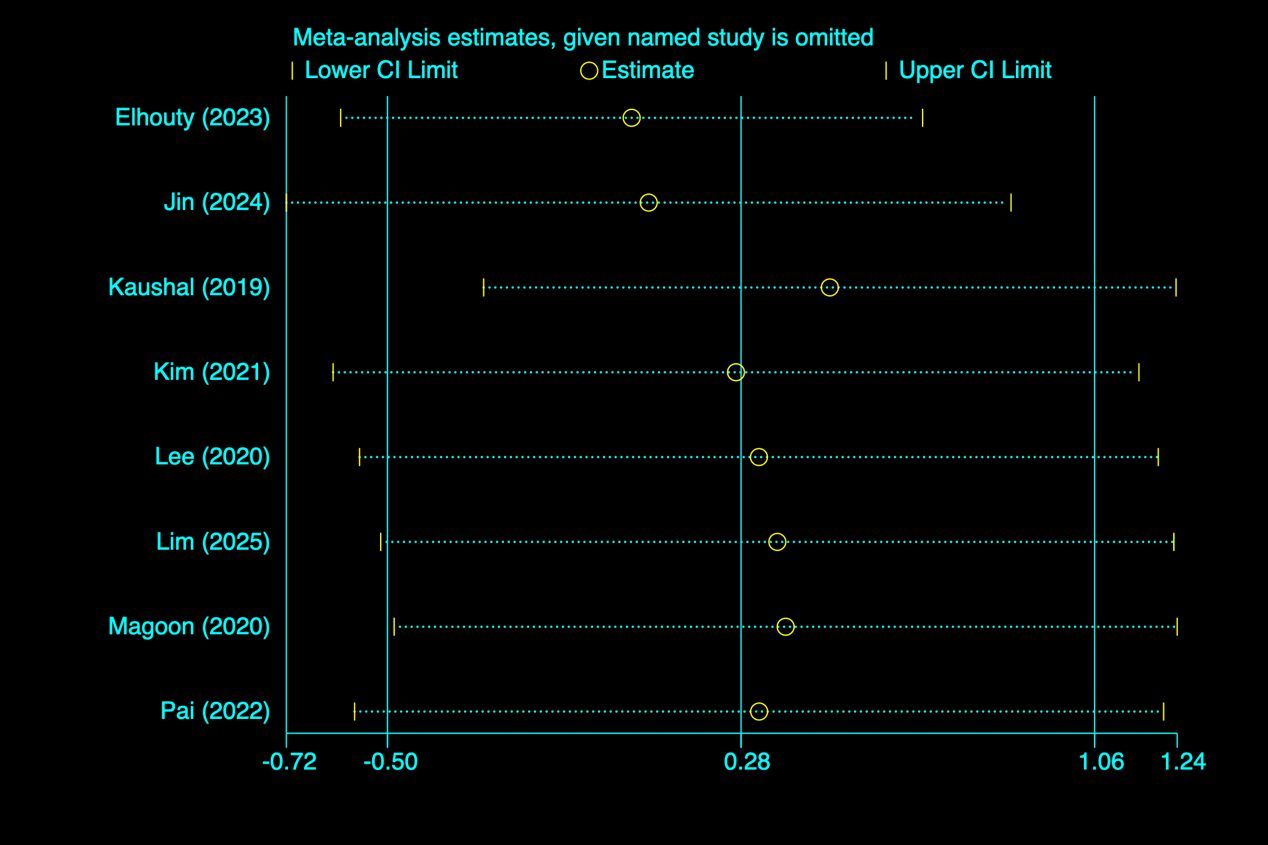


Figure S3 Sensitivity analysis of 6h pain score


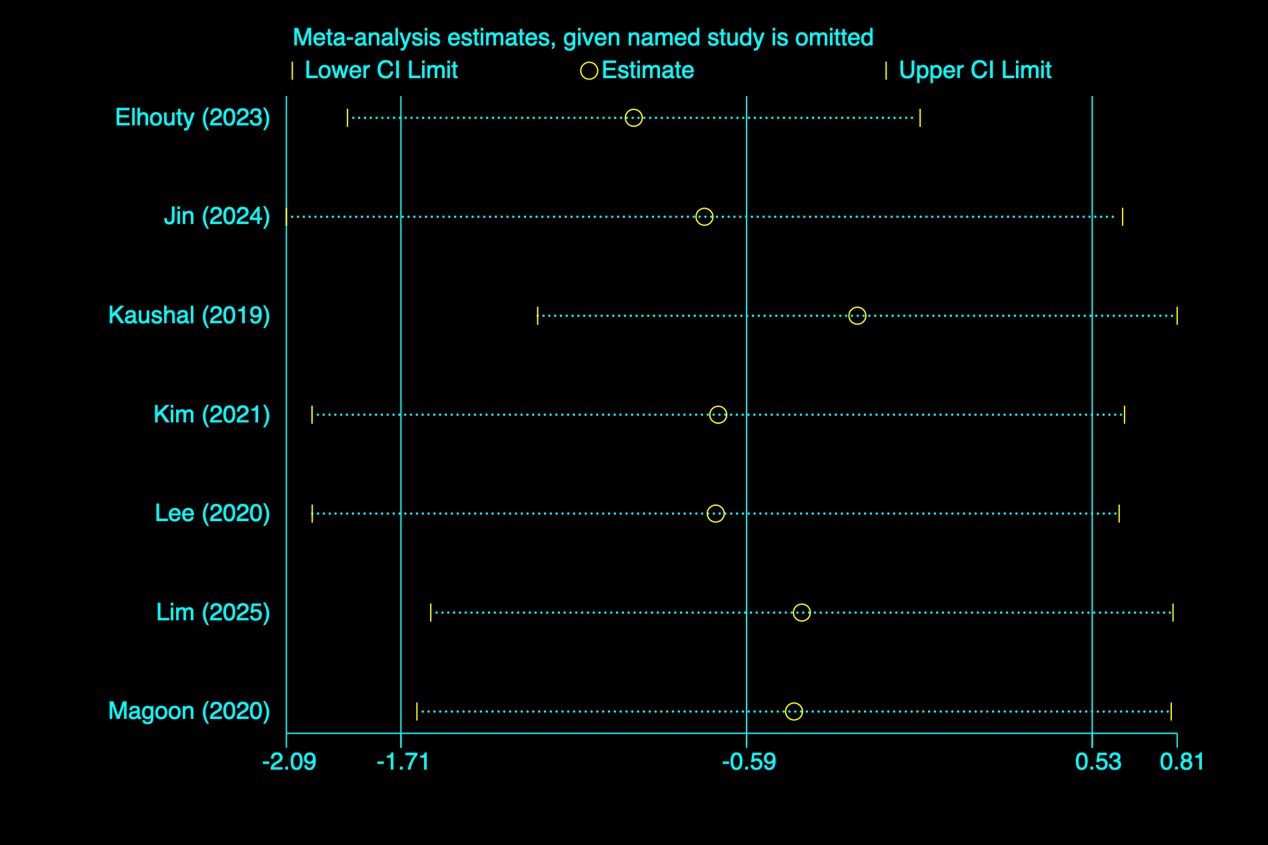


Figure S4 Sensitivity analysis of 12h pain score


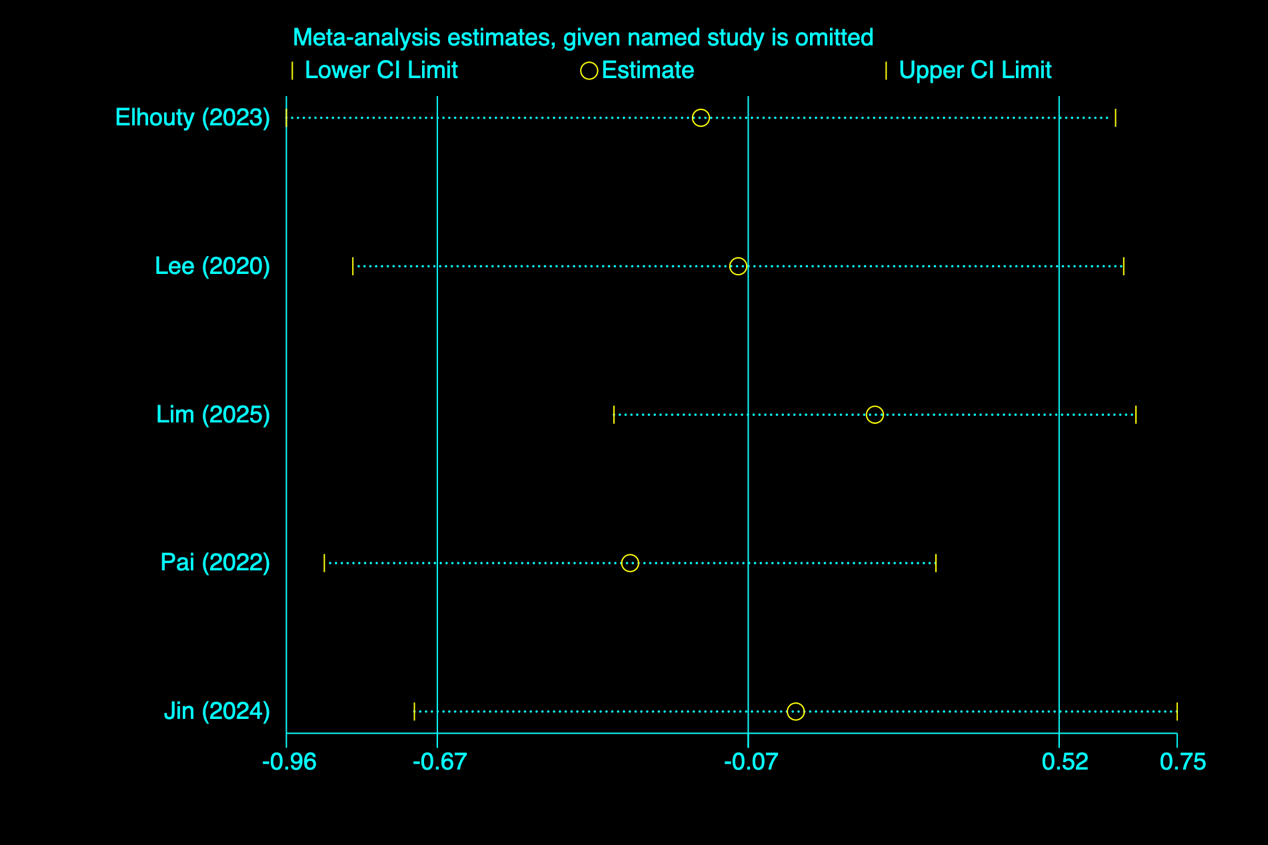


Figure S5 Sensitivity analysis of 24h pain score


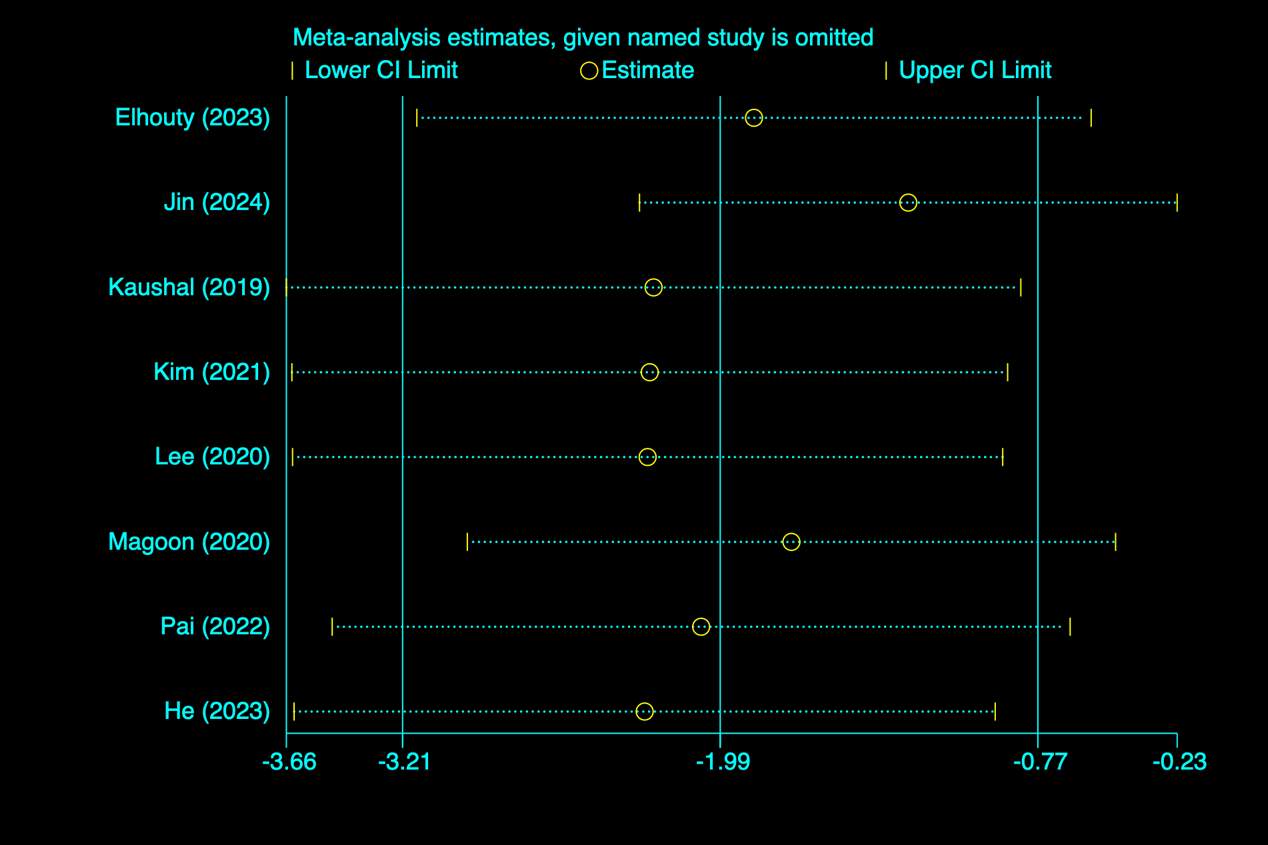


Figure S6 Sensitivity analysis of total opioid consumption


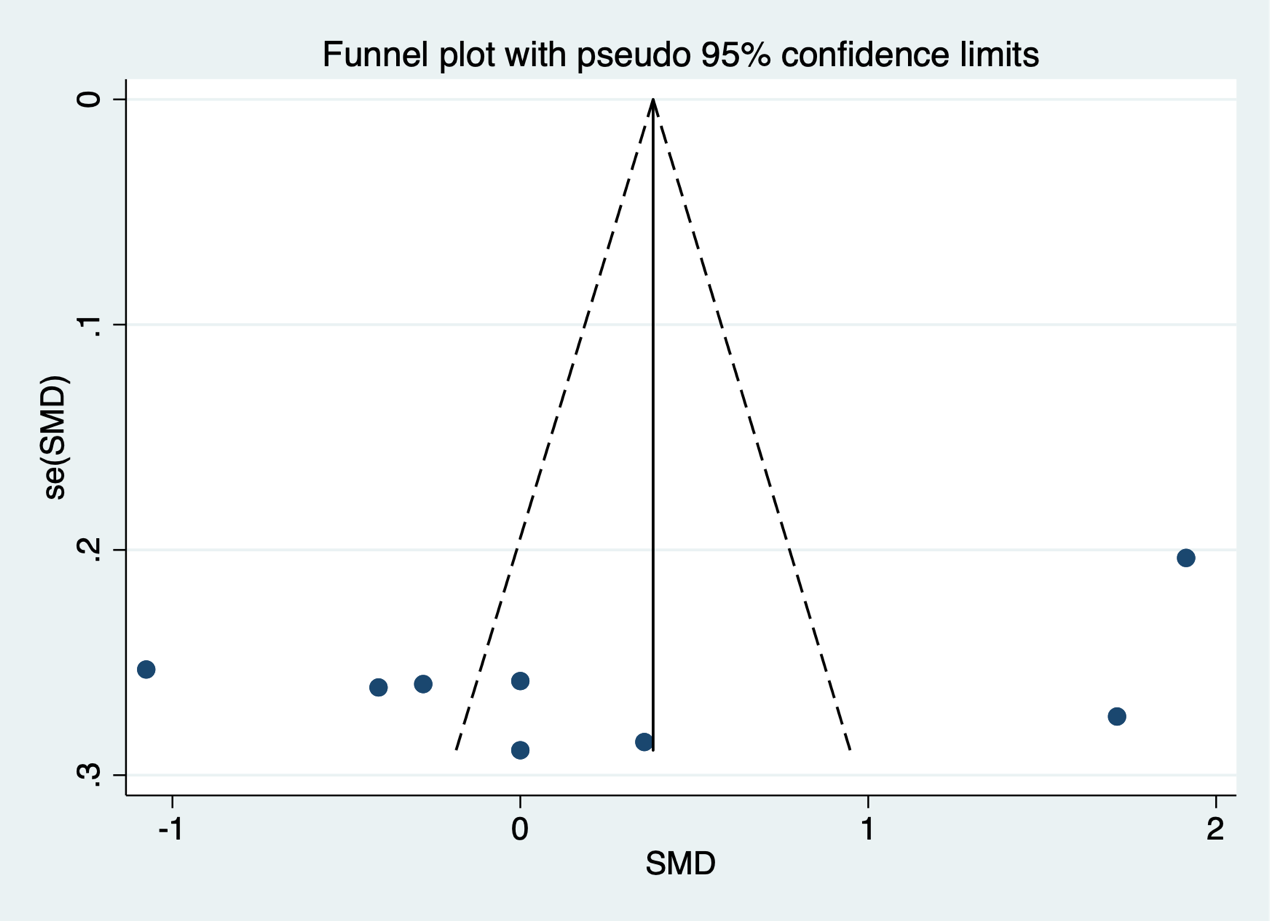


Figure S7 Funnel plot of the 6-hour pain scores meta-analysis


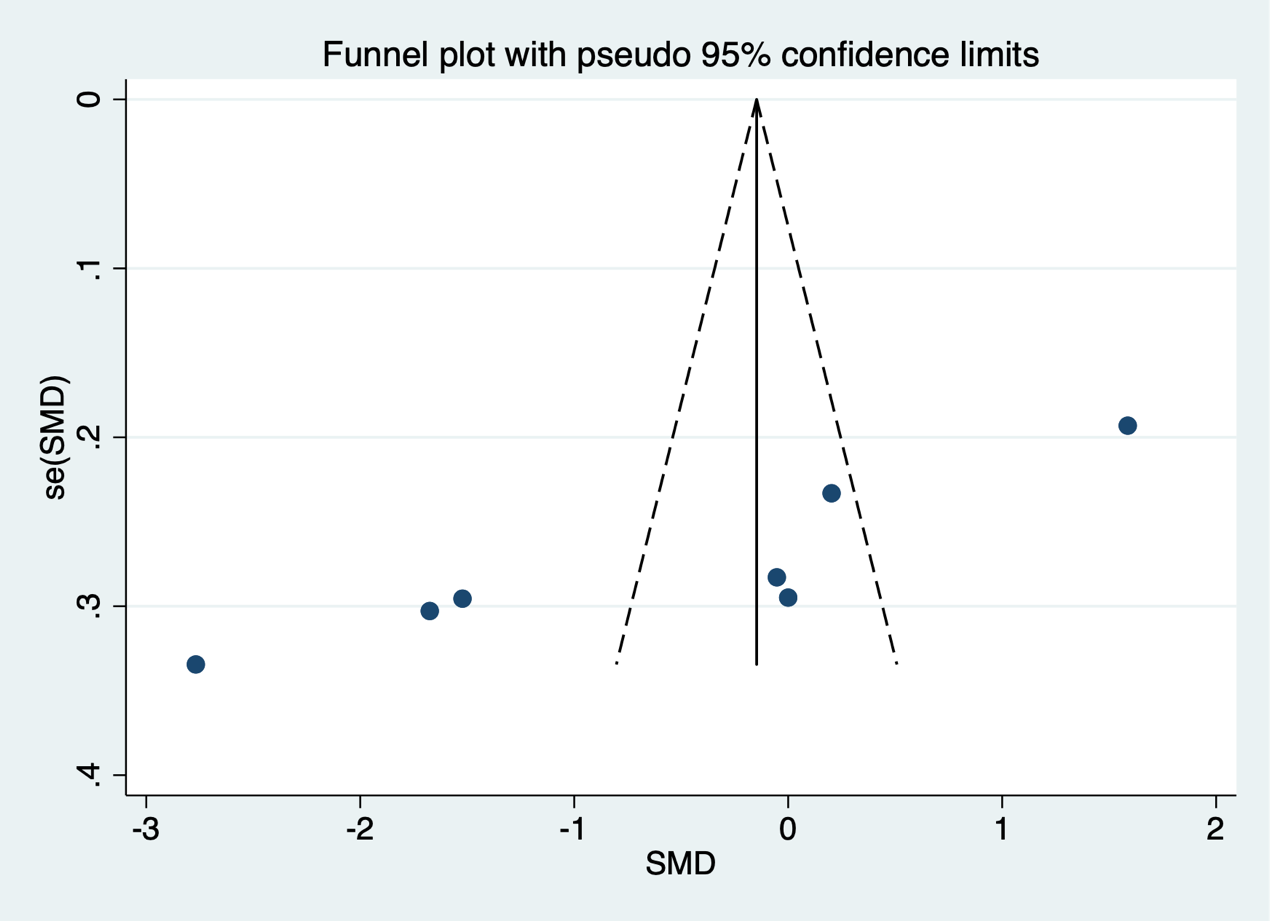


Figure S8 Funnel plot of the 12-hour pain scores meta-analysis


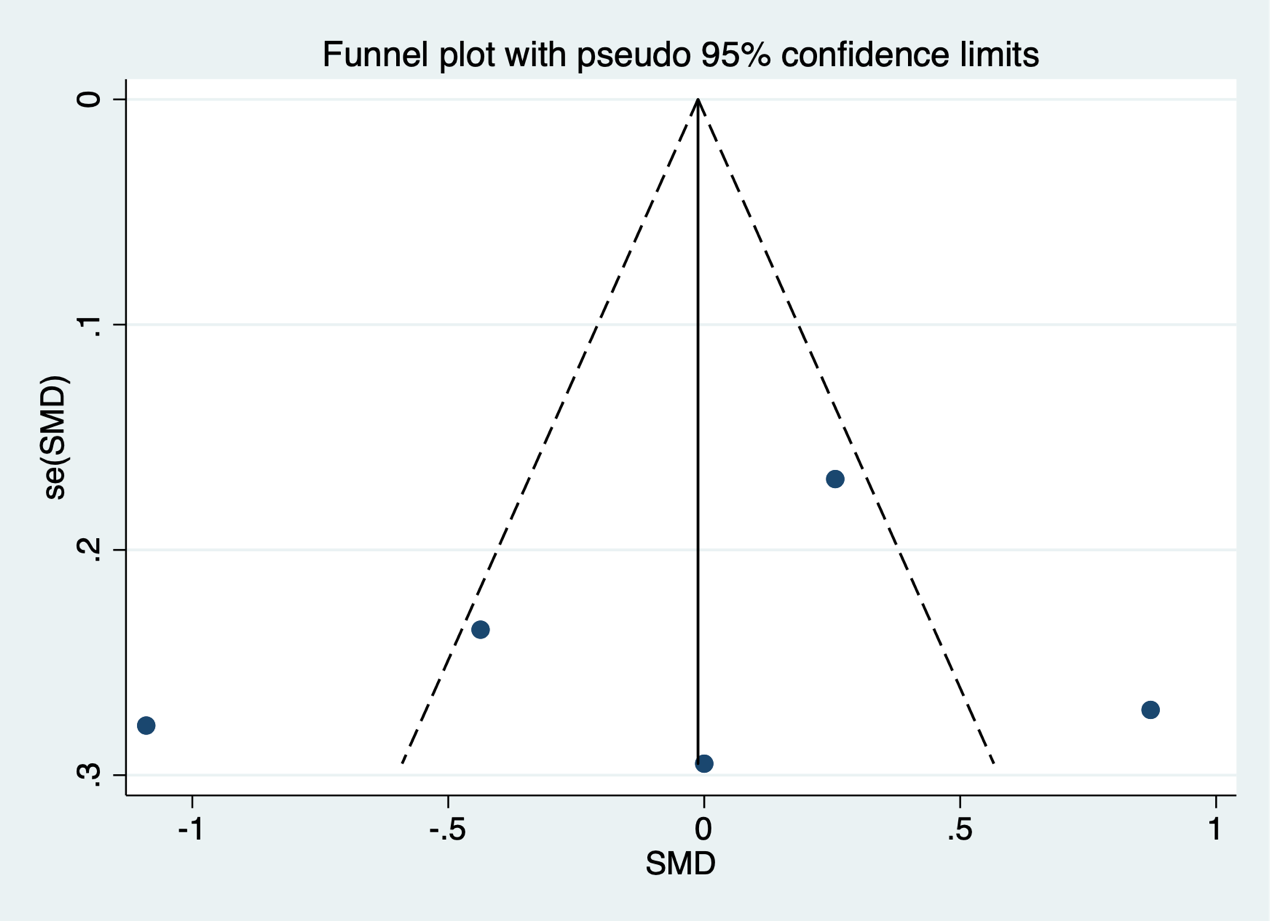


Figure S9 Funnel plot of the 24-hour pain scores meta-analysis


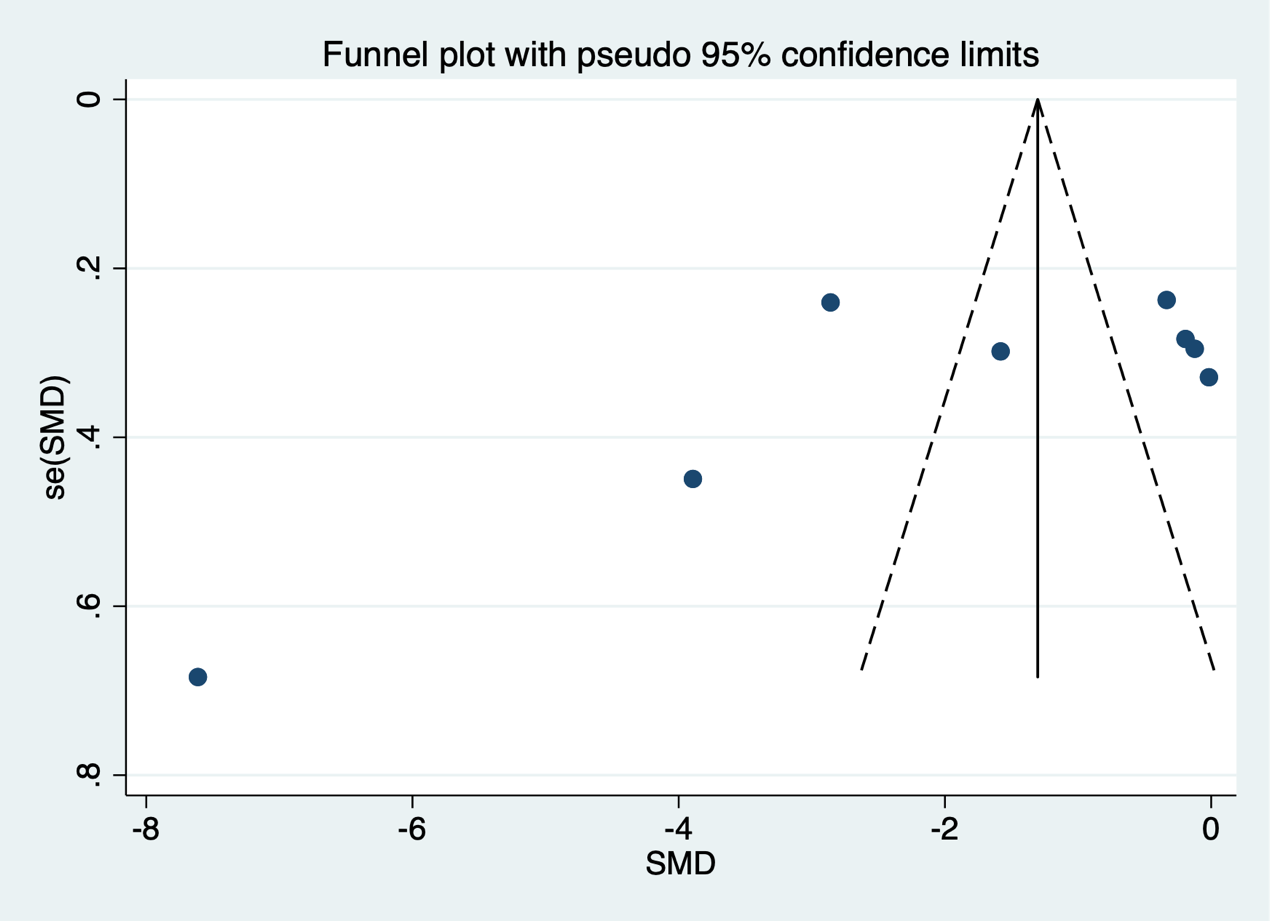


Figure S10 Funnel plot of total opioid consumption meta-analysis
